# Lycopene Promotes Osteogenesis and Reduces Adipogenesis through Regulating FoxO1/PPARγ Signaling in Ovariectomized Rats and Bone Marrow Mesenchymal Stem Cells

**DOI:** 10.3390/nu16101443

**Published:** 2024-05-10

**Authors:** Bingke Xia, Xuan Dai, Hanfen Shi, Jiyuan Yin, Tianshu Xu, Tianyuan Liu, Gaiyue Yue, Haochen Guo, Ruiqiong Liang, Yage Liu, Junfeng Gao, Xinxiang Wang, Xiaofei Chen, Jinfa Tang, Lili Wang, Ruyuan Zhu, Dongwei Zhang

**Affiliations:** 1 Diabetes Research Center, Traditional Chinese Medicine School, Beijing University of Chinese Medicine, Beijing 100029, China; xiabk1230@163.com (B.X.); dx15077872512@163.com (X.D.); shf1005@bucm.edu.cn (H.S.); yinjiyuan959@163.com (J.Y.); xuts1999@163.com (T.X.); liutianyuanlty@126.com (T.L.); yueyue@163.com (G.Y.); bucm18234610080@163.com (H.G.); l19800306030@163.com (R.L.); liuyage103@163.com (Y.L.); 2 Food and Pharmacy College, Xuchang University, 88 Bayi Road, Xuchang 461000, China; 3 The Scientific Research Center, Dongfang Hospital, Beijing University of Chinese Medicine, Beijing 100078, China; gaojunfeng860407@sohu.com (J.G.); wangxinxiangcn@aliyun.com (X.W.); 4 Department of Pharmacology, The First Affiliated Hospital of Henan University of Chinese Medicine, Zhengzhou 450003, China; chenxiaofei5555@163.com (X.C.); a0519@163.com (J.T.); 5 Department of TCM Pharmacology, Chinese Material Medica School, Beijing University of Chinese Medicine, Beijing 102488, China; 6 Institute of Basic Theory for Chinese Medicine, China Academy of Chinese Medical Sciences, Beijing 100700, China

**Keywords:** lycopene, OVX rats, BMSCs, osteogenesis, adipogenesis, FoxO1/PPARγ signaling

## Abstract

Recent interest in preventing the development of osteoporosis has focused on the regulation of redox homeostasis. However, the action of lycopene (LYC), a strong natural antioxidant compound, on osteoporotic bone loss remains largely unknown. Here, we show that oral administration of LYC to OVX rats for 12 weeks reduced body weight gain, improved lipid metabolism, and preserved bone quality. In addition, LYC treatment inhibited ROS overgeneration in serum and bone marrow in OVX rats, and in BMSCs upon H_2_O_2_ stimulation, leading to inhibiting adipogenesis and promoting osteogenesis during bone remodeling. At the molecular level, LYC improved bone quality via an increase in the expressions of FoxO1 and Runx2 and a decrease in the expressions of PPARγ and C/EBPα in OVX rats and BMSCs. Collectively, these findings suggest that LYC attenuates osteoporotic bone loss through promoting osteogenesis and inhibiting adipogenesis via regulation of the FoxO1/PPARγ pathway driven by oxidative stress, presenting a novel strategy for osteoporosis management.

## 1. Introduction

Lycopene (LYC), one kind of dietary lipid-soluble carotenoids, which is mostly found in tomatoes and other fruits with red color [1], and is well known for its high antioxidant potential [2]. As a natural nutrient, LYC is appealing to scientists and clinicians’ interests for its great contribution to attenuating various disorders such as cancers [3,4], cardiovascular disease [5], aging [6], obesity and diabetes [7]. Interestingly, recent findings have suggested that LYC may improve bone quality and attenuate bone loss in obese and osteoporotic animals [8,9]. However, the underlying mechanisms of this compound on osteoporosis still need further investigation.

Osteoporosis is a kind of degenerative bone diseases characterized by skeletal fragility and microarchitectural deterioration, leading to an increased risk of fracture [10,11]. Epidemiological evidence suggests that the prevalence of osteoporosis has continuously increased over the past decades [12]. The consequent high risk of disability and mortality of this disease has become one of the major threats to life expectancy and quality in the aging population [13], considering the undesired side effects and limitations of the current anti-osteoporotic medications [9,14]. Thus, clinical trials are still waiting for the new countermeasures to the treatment of osteoporosis.

The forkhead transcription factor O 1 (FoxO1), a conserved transcriptional factor [15], is abundantly expressed in the bones, liver and adipose tissues [16] with the function of regulating energy metabolism and oxidative stress [17,18]. In particular, FoxO1 is highly expressed in osteoblasts and involved in the development of bone metabolic diseases [18]. There is emerging evidence suggests that upregulation of FoxO1 expression alleviates bone quality through promoting adipogenesis and suppressing osteogenesis via inhibition of PPARγ expression in bone marrow mesenchymal stem cells (BMSCs) [19,20]. In contrast, an increased expression of PPARγ triggered by oxidative stress may divert BMSCs from osteogenesis to adipogenesis [21], thus contributing to the development of osteoporosis [22].

We have previously demonstrated that LYC prevented the development of osteoporosis in obese mice [23]. In addition, we have also found that LYC improved lipid metabolism in obese mice [24]. The evidence from clinical studies and preclinical trials suggests that LYC may have a beneficial role in the management of osteoporosis [25,26,27]. In light of these findings, we hypothesize that LYC may improve bone remodeling through the regulation of osteogenesis and adipogenesis to prevent the development of osteoporosis. For this purpose, ovariectomized (OVX) rats and BMSCs were used to investigate the actions and mechanisms of LYC on bone quality. 

## 2. Materials and Methods

### 2.1. Materials

LYC was purchased from RuiFenSi Biotechnology Co., Ltd. (Chengdu, China). Alizarin Red S was purchased from the Sigma-Aldrich (St. Louis, MO, USA). Antigen retrieval solution was bought from the ShunBai Biotechnology Company (No: SBT10013; Shanghai, China). The kits for triacylglycerols (TAGs), total cholesterol (TC), high-density lipoprotein (HDL), low-density lipoprotein (LDL), total antioxidant capacity (T-AOC), superoxide dismutase (SOD), malondialdehyde (MDA) were purchased from the Nanjing Jiancheng Bioengineering Institute (Nanjing, China). The ELISA kits, including N-terminal propeptide of type 1 procollagen (P1NP; Cat#: MB-7402A) and C-terminal cross-linked telopeptide of type I collagen (CTX-1; Cat#: MB-7275A) were purchased from the Jiangsu MeiBiao Biological Technology Co., Ltd. (Yancheng, China). Antibodies against OCN (1:500, Cat#: WLH4378) and Runx2 (1:1000, Cat#: WL03358) were bought from Wanlei Biotechnology (Shenyang, China). Antibodies against FoxO1 (1:3000, Cat#: 18592-AP), PPARγ (1:2000, Cat#: 16643-1-AP), C/EBPα (1:1000, Cat#: 18311-1-AP), GAPDH (1:10,000, Cat#: 60004-1-Ig), β-actin (1:10,000, Cat#: 66009-1-Ig) and HRP-conjugated goat anti-rabbit (1:12,000, Cat#: SA00001-4) and anti-mouse (1:12,000, Cat#: SA00001-1) secondary antibodies were from the Proteintech Biotechnology (Wuhan, China). HRP-conjugated goat anti-rabbit and anti-mouse secondary antibodies were procured from the Proteintech Biotechnology. All other reagents, except those specially identified, were from the Sinopharm Reagents Co., Ltd. (Beijing, China).

### 2.2. BMSCs Culture

BMSCs were isolated and identified from the femurs and tibias of 80–100 g Sprague-Dawley (SD) rats according to the procedures provided in the previous publication [28]. Briefly, the rat was killed by cervical dislocation and immersed in 75% ethanol for about 10 min. Then, both the femurs and tibias were removed from the animal body. And the bone marrow was rinsed with DMEM/low glucose medium using a syringe. After centrifugation, the pellets were collected and cultured with DMEM/low glucose medium containing 10% fetal bovine serum (FBS) and 1% penicillin/streptomycin in a humidified incubator containing 5% CO_2_ at 37 °C. BMSCs less than 3 passages were used for further experiments [29]. For characterization of BMSCs, 5 × 10^6^ BMSCs were collected and washed with PBS. Then the cells were incubated with PE-labeled anti-CD90 and APC-labeled anti-CD45 at 4 °C for 40 min, and subsequently subjected to flow cytometry assay [30]. The in vitro experiment was conducted from September 2020 to December 2022.

### 2.3. Cell Viability Assay

BMSCs (1 × 10^4^ cells) were cultured in a 96-well plate until approximately 70% confluence for CCK8 analysis. Then, cells were treated with various concentrations of LYC (1–10 μM) for 24, 48 and 72 h, respectively [31]. Subsequently, serum-free medium with 10% CCK8 was added to each well of the plate. And absorbance at 450 nm was measured using a FLUOstar Omega microplate reader (BMG LABTECH, Offenburg, Germany) after incubation at 37 °C for 1 h. For each condition, six replicates were performed throughout the experiments.

### 2.4. Intracellular ROS Examination

ROS production was measured using a 2,7-dichlorofluorescein diacetate (DCFH-DA) fluorescent probe [32]. For observation of DCF fluorescence, 1 × 10^4^ BMSCs were seeded in 96-well black plates and cultured with DMEM/low glucose medium for 24 h. Then, the cells were cultured with LYC and/or osteogenic induction medium for 24 h. Subsequently, cells were treated with 200 μM H_2_O_2_ for 2 h and followed by incubation with 10 μM DCFH-DA at 37 °C for 20 min in the dark. Finally, cells were washed with a serum-free medium. And the fluorescence intensity was measured under the automatic microplate reader.

### 2.5. Alizarin Red S Staining

Alizarin Red S staining was used to evaluate the osteogenesis in BMSCs and bone tissues. For the staining, BMSCs were incubated with or without LYC in the presence of osteogenic induction medium for 14 days, and subsequently treated with 200 μM H_2_O_2_ for 2 h. Then, cells were fixed with 95% ethanol for 10 min at room temperature followed by staining with Alizarin Red S solution (1 g of Alizarin Red in 100 mL of distilled water, pH 4.0–4.2) for 0.5 h in the dark. After the staining, cells were washed with PBS and then photographed under a microscope (BX53, Olympus, Tokyo, Japan).

For bone tissues, Alizarin Red S staining was performed according to the protocol as previously published [33]. After staining, the slides were observed and photographed using a microscope. For the analysis, the relative interest of density of Alizarin red S staining was quantified using the Image Pro Plus software (V6.0, Media Cybernetics, Rockville, MD, USA).

### 2.6. Oil Red O (ORO) Staining

ORO staining was used to measure the lipids production in BMSCs. Briefly, after incubation with or without LYC in the presence of osteogenic induction medium for 14 days, BMSCs were treated with 200 μM H_2_O_2_ for 2 h, followed by fixation with 4% paraformaldehyde for 10 min at room temperature and staining with ORO solution (the stock solution (0.5 g of ORO in 100 mL of isopropanol) diluted with water (60:40, *v*/*v*)) for 0.5 h in the dark. After staining, cells were photographed under a microscope to examine the lipid production. And the relative interest of density of ORO staining was quantified using the Image Pro Plus 6.0 software.

### 2.7. Induction of Osteoporotic Models and LYC Administration

Fifty female SD rats (230 ± 10 g, 11 weeks of age) were purchased from the Beijing Jinmuyang Experimental Animal Breeding Co., Ltd., Beijing, China [certification number SCXK 2016-0010] and housed in the clean level animal facilities at the Beijing University of Chinese Medicine (BUCM) with the temperature of 22 ± 1 °C, humidity of 55 ± 5% and a 12-h light/dark cycle. All the rats were allowed free access to tap water and chow. All the animal protocols were approved by the Animal Care Committee of BUCM, China (Protocol code: BUCM-4-2019061701-3001; Date of approval: 17 June 2019).

After acclimation for 1 week, rats were anesthetized and ovariectomized by removing the bilateral ovaries to establish osteoporotic models according to the previous procedures [34]. In addition, sham-operated rats were subjected to a similar operation by removing an equal volume of fats surrounding the ovaries [35].

**Figure 1:**
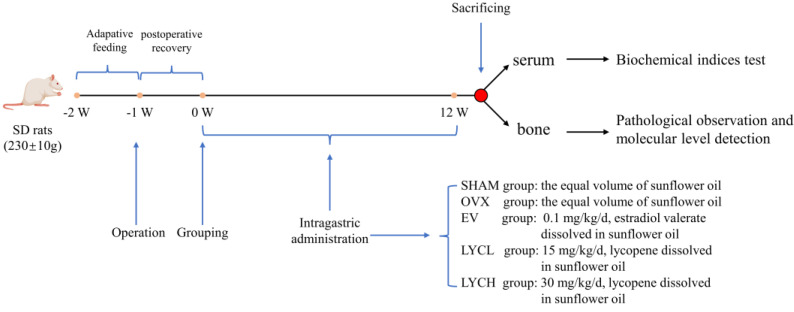
The flow chart for animal experiments.

One week after surgery, ovariectomized rats were randomly divided into 4 groups with 10 in each, namely the OVX, estradiol valerate (EV), high-dose LYC (LYCH) and low-dose LYC (LYCL) groups (Figure 1). Rats in the EV, LYCH and LYCL groups were orally administrated with estradiol valerate tablets (0.1 mg/kg), and LYC (LYCH, 30 mg/kg; LYCL, 15 mg/kg) dissolved in sunflower oil, respectively. Rats in the OVX and SHAM groups were orally gavaged with an equal volume of the vehicle. During the treatment, body weight was recorded every week. After 12 weeks of intervention, serum was collected from the abdominal aorta of anesthetized rats. Then, the uterus was removed and weighed. And the bilateral tibias and femurs were dissected from the animal body. The samples were then either stored at −80 °C or soaked in 10% neutral formalin for further experiments. The in vivo experiments were performed from June 2019 to August 2021.

### 2.8. Serum and Bone Marrow Biomarkers Analysis

Serum TAGs, TC, HDL, LDL, bone marrow TAGs, and TC were detected using the corresponding commercial kits according to the manufacturer’s instructions. The levels of T-AOC, SOD and MDA were determined by biochemical assays. The serum levels of CTX-1 and P1NP were also measured using the corresponding ELISA kits according to the manufacturer’s instructions.

### 2.9. μ-CT Scanning

The right femurs were subjected to the μ-CT scanning as previously described [36,37]. Briefly, the right femur was scanned and captured by the Quantum GX μCT instrument (PerkinElmer, Waltham, MA, USA). The parameters in the volume of interest were analyzed by the Analyzer Software (V12.0), including (1) BMD (bone mineral density); (2) BV/TV (bone volume fraction); (3) BS/TV (bone surface density); (4) Tb.N (trabecular number); (5) Tb.Sp (trabecular separation); (6) Tb.Th (trabecular thickness); (7) Conn.D (connectivity density); and (8) SMI (Structure Model Index).

### 2.10. Bone Biomechanical Strength Assay

After the μCT scanning, the right femurs were taken for a three-point bending assay by an electronic universal testing machine (AGS-X500, Shimadzu, Kyoto, Japan), as previously described [36,37]. The shaft of the femur was fixed between the two supporting points, with a distance of 20 mm. Then, a certain load was vertically administered to the tibial midshaft at the speed of 1 mm/min until the tibial shaft is fractured. The ultimate load, bending strength, and elastic modulus of the femurs were analyzed by an electronic universal testing apparatus.

### 2.11. Fourier Transform Infrared Spectroscopy (FTIR) Assay

After that, the rat femur was ground to powder in a ceramic mortar. The spectrum was obtained by the FTIR (Vertex 70, Bruker, Karlsruhe, Germany). Scanning was performed in transmission mode in the 4000–400 cm^−1^ range with accumulating 64 scans. The relative ratio of carbonate to phosphate, the area ratio v1, v3 band to v2 CO_3_ were determined as previously published [38,39].

### 2.12. Hematoxylin & Eosin (H&E) and Safranin O-Fast Green Staining

The left femurs of the rats were fixed with 10% neutral formalin and then decalcified in 10% neutral EDTA buffer for 3 months. Then, the femurs were embedded in paraffin. Sections (5-µm) were subjected to the H&E staining and safranin O-fast green staining according to the routine protocols [33,40].

### 2.13. Immunohistochemistry (IHC) Staining

IHC staining was conducted according to the procedures provided in the previous publication [36]. Briefly, a 5-μm section was incubated with appropriate primary antibody [OCN (1:1000), Runx2 (1:1000), PPARγ (1:1000) and FoxO1 (1:1000)], respectively, overnight at 4 °C. Subsequently, the section was incubated with the corresponding horseradish peroxidase-conjugated secondary antibody. Finally, the section was observed and photographed using an Olympus BX53 microscopy. The intensity of positive staining was analyzed using the Image Pro Plus 6.0 software and expressed as an IOD value.

### 2.14. Western Blot Assay

Proteins were obtained from the femurs and BMSCs, and determined using a BCA assay kit. After that, the proteins were subjected to SDS-PAGE gel and transferred onto PVDF membranes [29]. Then, the PVDF membranes were sequentially incubated with the appropriate primary antibody (Runx2, PPARγ, FoxO1, GAPDH, β-actin, and Lamin B1), respectively, at 4 °C overnight. The next day, after incubation with corresponding HRP-labeled secondary antibody for 1 h at room temperature, immune-positive bands were detected using high-sensitivity ECL and captured with Azure Bio-imaging systems (Azure Biosystems, Dublin, CA, USA). Gray values of the images were analyzed with Image J software (V1.51j8, NIH, Bethesda, MD, USA) and normalized with the same membrane of β-actin or Lamin B1 as the internal control.

### 2.15. Statistical Analysis

Data were analyzed by ANOVA or a nonparametric test according to the homogeneity of variance and normality (GraphPad Prism V8.3.0, Boston, MA, USA). Otherwise, LSD or Dunnett-t tests were conducted between multiple groups. The results were expressed as mean ± SD. *p* < 0.05 was considered as a statistical difference.

## 3. Results

### 3.1. LYC Preserves Bone Micro-Architecture, Strength, Material Properties in OVX Rats

**Figure 2:**
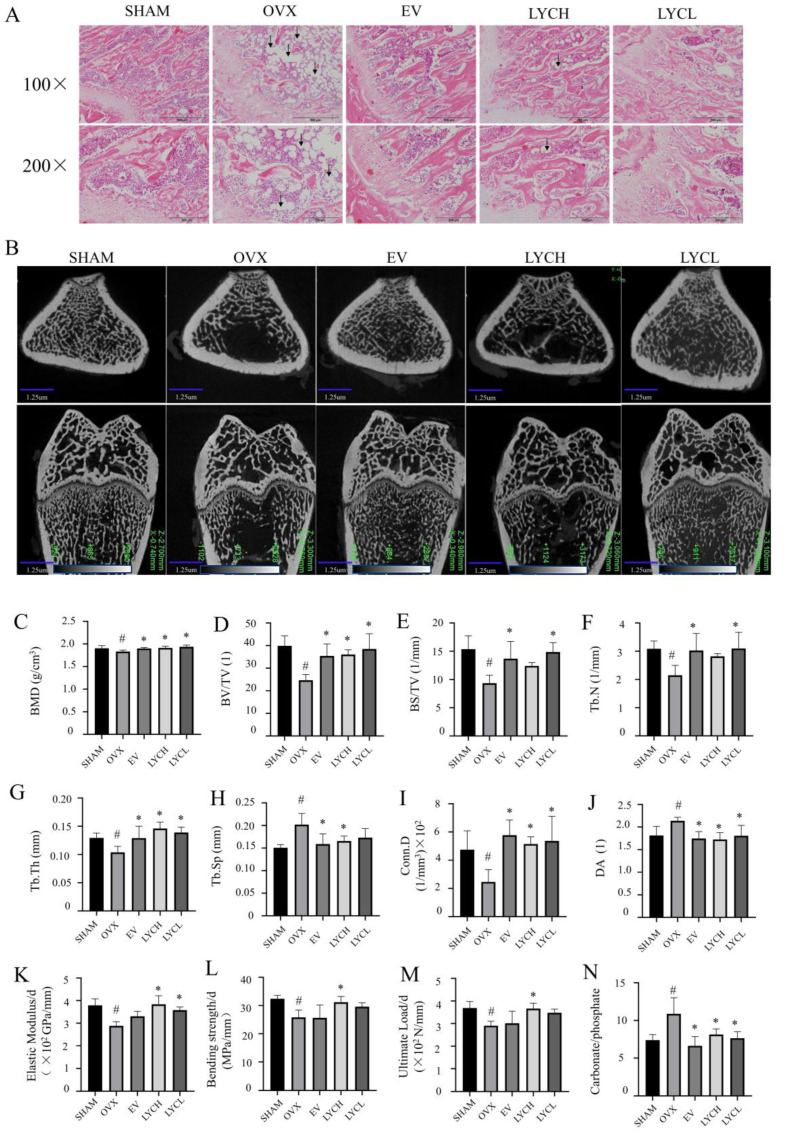
Lycopene preserves bone micro-architecture, strength, and material properties in OVX rats. Representative images of H&E (**A**) and μCT scanning (**B**) in the femurs of the different groups of rats. The BMD (**C**), BV/TV (**D**), BS/TV (1/mm) (**E**), Tb.N (1/mm) (**F**), Tb.Th (mm) (**G**), Tb.Sp (mm) (**H**), Conn.D (1/mm^3^) (**I**), and DA (**J**) in the femoral metaphysis were analyzed by the Analyzer Software. Elastic Modulus/d (**K**), Bending strength/d (**L**), Ultimate Load/d (**M**), and carbonate to phosphate (**N**) were determined by a three-point bending assay and Fourier-Transform Infrared Spectroscopy (FTIR), respectively. Data are presented as mean ± SD. The black arrow in panel (**A**) denotes lipid droplet. SHAM denotes the sham operation group, OVX denotes the ovariectomized model group, EV denotes the estradiol treatment group, LYCH denotes the high-dose lycopene treatment group, and LYCL denotes the low-dose lycopene treatment group. *n* = 5, ^#^ vs. the SHAM group, * vs. the OVX group. *p* < 0.05 was considered statistically significant.

As shown in Figure 2A, H&E staining revealed that the trabecular bone in the distal femurs of the SHAM group was orderly distributed and appeared normal meshwork. By contrast, the trabecular bone in the OVX group became thinner, and irregular and showed a disorganized mesh structure. In addition, the lipid droplets (indicated by the black arrow) were much more obvious in the femurs of the OVX group than those in the SHAM group. Interestingly, the trabecular bone and the number of lipid droplets of the LYC and EV group were almost returned to normal levels and similar to those in the SHAM group.

In order to further investigate the effect of LYC on the alterations of bone microstructure, the femurs were subjected to μCT scanning. The results of the μCT showed a significant decrease in bone volume and trabeculae numbers in the distal femurs of the OVX group relative to the SHAM group (Figure 2B). In detail, as shown in Figure 2C–J, the structural parameters of the distal femurs, including BMD, BV/TV, BS/TV, Tb.N, Tb.Th, Conn.D, Tb.Sp and DA were decreased and increased, respectively, in the OVX group compared with those in the SHAM group. However, treatment of OVX rats with EV or LYC for 12 weeks markedly improved the trabecular bone microstructure and reversed the abovementioned parameters (*p* < 0.05).

Next, we analyzed bone biomechanical properties, including elastic modulus, bending strength, and ultimate load in the femurs by three-point bending assay. As shown in Figure 2K–M, the elastic modulus, bending strength, and ultimate load in the femurs of the OVX group were significantly lower than those in the SHAM one (*p* < 0.05). As expected, treatment of OVX rats with EV or LYC prevented a decline in the abovementioned parameters in the femurs (*p* < 0.05).

To further evaluate the effect of LYC on bone material quality, the femurs were subjected to the FTIR. As shown in Figure 2N, the relative ratios of carbonate to phosphate were significantly increased in the femurs of the OVX group compared with those in the SHAM group (*p* < 0.05). While treatment with EV or LYC obviously reversed the alterations in the femurs of OVX rats (*p* < 0.05). These results suggest that LYC has the ability of improving bone strength and microstructure as well as preserving bone material profiles in OVX rats.

### 3.2. LYC Inhibits Oxidative Stress in OVX Rats and in BMSCs

**Figure 3:**
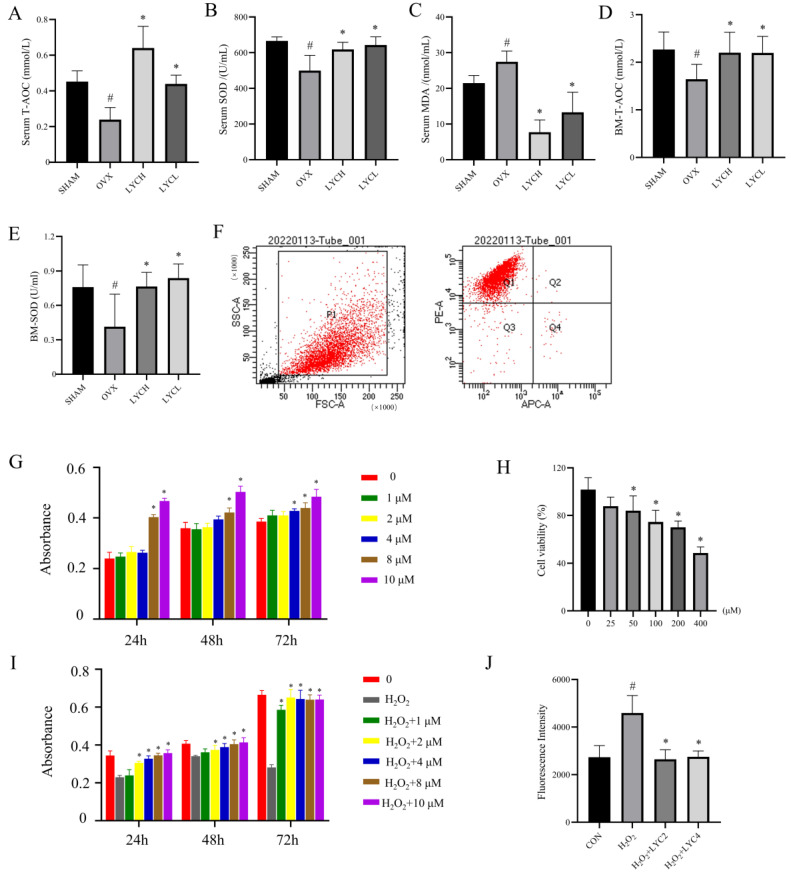
Lycopene inhibits oxidative stress in OVX rats and in BMSCs. Serum and bone marrow levels of oxidative stress markers were determined by biochemical assays, including serum total antioxidant capacity (Serum T-AOC, (**A**)), serum superoxide dismutase (Serum SOD, (**B**)), malondialdehyde (Serum MDA, (**C**)), bone marrow total antioxidant capacity (BM-T-AOC, (**D**)) and bone marrow superoxide dismutase (BM-SOD, (**E**)). The primary BMSCs were characterized by flow cytometry (**F**). Effects of lycopene on BMSCs proliferation with/without H_2_O_2_ exposure after 24, 48, and 72 h (**G**–**I**) were determined by a CCK-8 assay. The intracellular levels of ROS were determined by the DCFH-DA (**J**). SHAM denotes the sham operation group, OVX denotes the ovariectomized model group, EV denotes the estradiol treatment group, LYCH denotes the high-dose lycopene treatment group, LYCL denotes the low-dose lycopene treatment group. CON denotes the blank control, H_2_O_2_ denotes the stimulation with H_2_O_2_ for 1 h, LYC2 denotes the 2 μM of lycopene treatment, LYC4 denotes 4 μM of lycopene treatment. *n* = 5, ^#^ vs. the SHAM or CON group, * vs. the OVX or H_2_O_2_ group. *p* < 0.05 was considered statistically significant.

Inspired by the notion that LYC is a strong anti-oxidant product [41], we first investigated the effect of this compound on redox stress in OVX rats. As shown in Figure 3A–C, serum levels of T-AOC and SOD were decreased, and serum levels of MDA were increased, respectively, in the rats of the OVX group, relative to those in the SHAM group (*p* < 0.05). In addition, we further determined the levels of T-AOC and SOD in the bone marrow. As shown in Figure 3D,E, the levels of T-AOC and SOD were decreased in the OVX group, which were in line with serum redox status. As expected, supplement of LYC to OVX rats reversed the alterations of the abovementioned alterations in serum and bone marrow in comparison with those of the vehicle-treated ones (*p* < 0.05). These results suggest that LYC may alleviate oxidative stress in OVX rats.

In order to further investigate the actions of LYC on the primary BMSCs upon H_2_O_2_ stimulation, we first characterized BMSCs by flow cytometry. As shown in Figure 3F, BMSCs were characterized by a positive staining with CD90 and a negative staining for CD45, indicating that BMSCs were successfully isolated. Next, the effects of LYC on cell proliferation were detected by the CCK-8 assay. As shown in Figure 3G, 1 to 4 μM of LYC did not affect cell proliferation at 24 h and 48 h, indicating that LYC at these concentrations does not affect cell viability.

After identification of the optimum condition for H_2_O_2_ exposure (200 μM, 1 h; Figure 3H), the effects of LYC (0, 1, 2, 4, 8, and 10 μM) on H_2_O_2_-stimulated BMSCs were evaluated by the CCK-8 assay. As shown in Figure 3I, LYC at the levels of 2–10 μM can promote cell proliferation at 24, 48 and 72 h. Comprehensively considering the abovementioned findings, 2 and 4 μM of this compound were selected as the optimum concentration for the ensuing experiments.

Next, we examined whether LYC could attenuate H_2_O_2_-induced ROS production in BMSCs by DCFH-DA staining. As shown in Figure 3J, H_2_O_2_ stimulation induced a significant increase in the fluorescence intensity in BMSCs (*p* < 0.05). As expected, the addition of LYC (2 and 4 μM) to BMSCs significantly decreased fluorescence intensity relative to those of the vehicle-treated (*p* < 0.05). These results demonstrated that LYC was capable of rebuilding redox homeostasis in BMSCs and OVX rats.

### 3.3. LYC Improves Lipid Metabolism in OVX Rats and BMSCs

**Figure 4:**
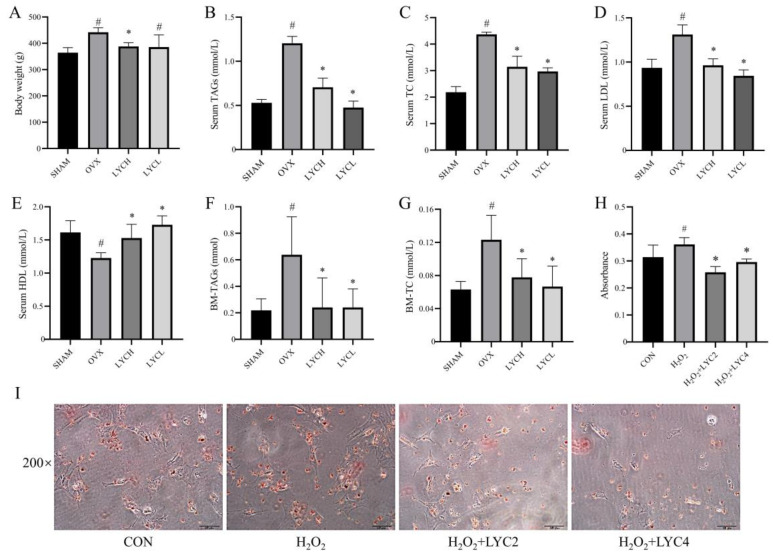
Lycopene improves lipid metabolism in OVX rats and in BMSCs. Body weight (**A**), serum triacylglycerols (Serum TAGs, (**B**)), serum total cholesterol (Serum TC, (**C**)), serum low-density lipoprotein (Serum LDL, (**D**)), serum high-density lipoprotein (Serum HDL, (**E**)), bone marrow TAGs (BM-TAGs, (**F**)) and bone marrow TC (BM-TC, (**G**)). The Oil Red O staining and its analysis in BMSCs (**I**,**H**). SHAM denotes the sham operation group, OVX denotes the ovariectomized model group, EV denotes the estradiol treatment group, LYCH denotes the high-dose lycopene treatment group, LYCL denotes the low-dose lycopene treatment group. CON denotes the blank control, H_2_O_2_ denotes the stimulation with H_2_O_2_ for 1 h, LYC2 denotes 2 μM of lycopene treatment, LYC4 denotes 4 μM of lycopene treatment. *n* = 5 (rats), *n* = 3 (cells), ^#^ vs. the SHAM or CON group, * vs. the OVX or H_2_O_2_ group. *p* < 0.05 was considered statistically significant.

As shown in Figure 4A, in the 12th week, the body weight of the rats in the OVX group was significantly greater than that in the SHAM group. However, compared to the OVX group, the body weight of the rats in the LYCL and LYCH groups was significantly decreased (*p* < 0.05). In addition, as shown in Figure 4B–E, the rats in the OVX group showed an obvious increase in serum levels of TAGs, TC, and LDL and a significant decrease in serum levels of HDL, respectively, relative to those in the SHAM group (*p* < 0.05). Interestingly, LYC (LYCL and LYCH) treatment reversed the abovementioned alterations in OVX rats (*p* < 0.05).

Next, we investigated the alterations of lipid profiles in the bone marrow. As shown in Figure 4F,G, the levels of TAGs and TC in the bone marrow of the OVX group were markedly increased relative to those in the SHAM group (*p* < 0.05). Similarly, LYC (LYCL and LYCH) treatment notably reversed the alterations in the bone marrow of OVX rats (*p* < 0.05). Additionally, as shown in Figure 4H,I, LYC administration significantly reduced the accumulation of lipid droplets in BMSCs under H_2_O_2_ stimulation (*p* < 0.05). These results suggest that LYC could improve lipid metabolism in OVX rats.

### 3.4. LYC Promotes Osteogenesis in OVX Rats and in BMSCs

**Figure 5:**
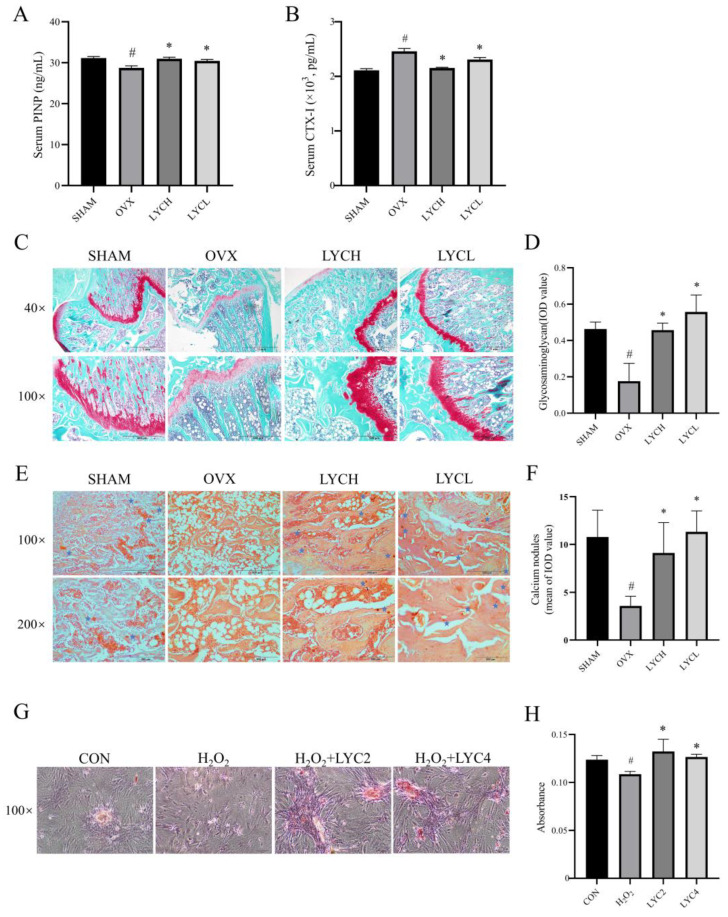
Lycopene promotes osteogenesis in OVX rats and in BMSCs. Serum levels of PINP (**A**) and CTX-I (**B**). (**C**,**D**) The representative images of Safranin O/fast green staining and their analyses show the GAG levels in the femurs. The representative images of alizarin red S staining and their analyses show the osteogenesis and calcium nodules in the different groups of rats (**E**,**F**) and BMSCs (**G**,**H**). Blue star denotes calcium nodules (**E**). SHAM denotes the sham operation group, OVX denotes the ovariectomized model group, EV denotes the estradiol treatment group, LYCH denotes the high-dose lycopene treatment group, LYCL denotes the low-dose lycopene treatment group. CON denotes the blank control, H_2_O_2_ denotes the stimulation with H_2_O_2_ for 1 h, LYC2 denotes 2 μM of lycopene treatment, LYC4 denotes 4 μM of lycopene treatment. *n* = 5 (rats), *n* = 3 (cells), ^#^ vs. the SHAM or CON group, * vs. the OVX or H_2_O_2_ group. *p* < 0.05 was considered statistically significant.

It is well known that serum P1NP is an indicator of the synthesis of type I collagen, and is positively correlated with bone formation. Serum CTX-1 is a product of type I collagen degradation, and is positively associated with bone resorption [42]. As shown in Figure 5A,B, the results from the ELISA demonstrated that serum P1NP levels and CTX-1 levels were significantly decreased and increased, respectively, in the OVX group when compared with those in the SHAM group (*p* < 0.05). However, after treatment with LYC (LYCL and LYCH) for 12 weeks, the alterations of serum P1NP and CTX-1 were notably reversed as compared to those of the vehicle-treated OVX ones (*p* < 0.05).

Glycosaminoglycans (GAGs) play a favorable role in bone remodeling through the promotion of osteogenesis [43]. And Safranin O dye stains proteoglycans in cartilage to red. As shown in Figure 5C,D, the results from Safranin O staining revealed that the GAGs levels were significantly reduced in the OVX group when compared to those in the SHAM group (*p* < 0.05). Not surprisingly, after treatment with LYC (LYCL and LYCH) for 12 weeks, the GAGs levels were significantly increased when compared to those in the OVX group (*p* < 0.05).

Next, we examined the effect of LYC on osteogenesis in OVX rats. The alizarin red S assay is considered the gold standard for evaluation of osteoblast mineralization. As shown in Figure 5E,F, the results from the Alizarin Red S staining (indicated by the blue pentagram) showed that the amount of calcium nodules was significantly reduced in the femurs of the OVX group compared to those in the SHAM group (*p* < 0.05). Notably, LYC (LYCL and LYCH) treatment obviously improved the distribution and increased the areas of calcium nodules in the femurs as compared to those of the vehicle-treated OVX ones (*p* < 0.05). Additionally, LYCL is better than LYCH in attenuating bone histomorphology disorders in the femurs of OVX rats (*p* < 0.05). Therefore, in the following experiments, LYCL was selected to further study the underlying mechanisms of this compound in the prevention of osteoporosis.

Moreover, as shown in Figure 5G,H, the calcium nodules were formed in BMSCs subjected to osteogenic induction in the CON group. H_2_O_2_ stimulation significantly reduced the formation of calcium nodules in BMSCs relative to those of the vehicle-treated ones (*p* < 0.05). Interestingly, the addition of LYC (2 and 4 μM) obviously attenuated the limitation of calcium nodule formation in BMSCs upon H_2_O_2_ stimulation (*p* < 0.05). These findings indicate that LYC may attenuate bone loss by promoting bone formation and inhibiting bone resorption in OVX rats.

### 3.5. LYC Increases FoxO1, Runx2, and OCN Expressions, and Inhibits PPARγ and C/EBPα Expressions in the Femurs and Tibias of OVX Rats and BMSCs

**Figure 6:**
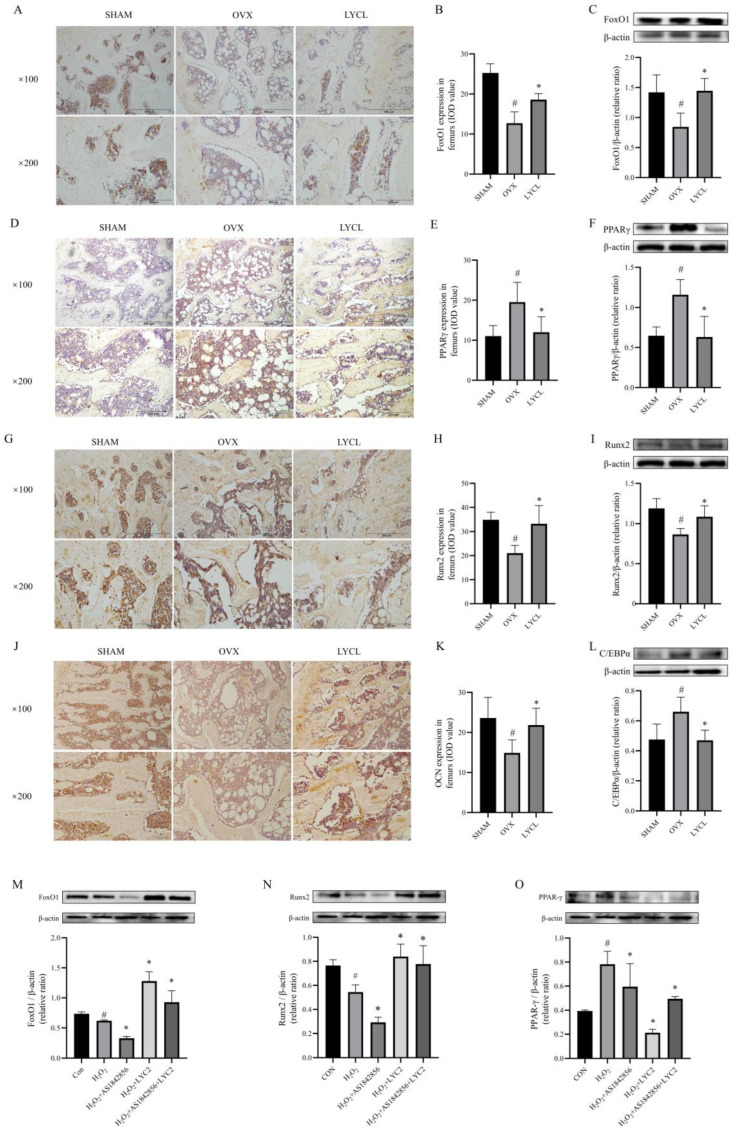
Lycopene increases FoxO1, Runx2, and OCN expressions, and inhibits PPARγ and C/EBPα expressions in the femurs and tibias of OVX rats and in BMSCs. The expressions of FoxO1, PPARγ, Runx2, OCN, and C/EBPα in the femurs and tibias were determined by immunohistochemical staining (**A**,**B**,**D**,**E**,**G**,**H**,**J**,**K**) and/or western blot (**C**,**F**,**I**,**L**). The expressions of FoxO1, Runx2, and PPARγ in BMSCs were determined by western blot (**M**–**O**). SHAM denotes the sham operation group, OVX denotes the ovariectomized model group, LYCL denotes the low-dose lycopene treatment group. CON denotes the blank control, H_2_O_2_ denotes the stimulation with H_2_O_2_ for 1 h, LYC2 denotes 2 μM of lycopene treatment, AS1842856 denotes FoxO1 inhibitor, AS1842856+LYC2 denotes the co-treatment of FoxO1 inhibitor and 2 μM of lycopene. *n* = 5 (rats), *n* = 3 (cells), ^#^ vs. the SHAM or CON group, * vs. the OVX or H_2_O_2_ group. *p* < 0.05 was considered statistically significant.

As shown in Figure 6A–C,G–K, the results from IHC staining showed that the expressions of FoxO1, Runx2, and OCN were significantly decreased in the femurs and tibias of the OVX group than those in the SHAM group (*p* < 0.05). As expected, LYC treatment significantly increased the expressions of FoxO1, Runx2, and OCN in the bones of OVX rats compared to those of the vehicle-treated ones (*p* < 0.05). In addition, similar alterations of the FoxO1 and Runx2 were observed from the results of western blots.

Moreover, as shown in Figure 6D–F,L, the expressions of PPARγ and C/EBPα by IHC staining and/or western blots were increased in the femurs and tibias of the OVX group in comparison with those in the SHAM group (*p* < 0.05). Intriguingly, LYC treatment markedly reduced the expressions of PPARγ and C/EBPα in the femurs and tibias of OVX rats (*p* < 0.05). These results suggest that LYC has the ability of promoting osteogenesis and suppressing adipogenesis in OVX rats.

In order to further elucidate the mechanism behind these alterations, BMSCs were subjected to H_2_O_2_, LYC and/or AS1842856 (a FoxO1 inhibitor) stimulation. As shown in Figure 6M,N, the results from western blots showed that the expressions of FoxO1 and Runx2 were notably decreased in BMSCs upon H_2_O_2_ and AS1842856 stimulation when compared to those of the vehicle-treated ones (*p* < 0.05). Interestingly, LYC treatment significantly reversed the expressions of FoxO1 and Runx2 in BMSCs upon H_2_O_2_ and/or AS1842856 exposure (*p* < 0.05).

In addition, as shown in Figure 6O, the expression of PPARγ in BMSCs was significantly increased upon H_2_O_2_ and AS1842856 stimulation compared with those of the vehicle-treated (*p* < 0.05). Intriguingly, this alteration was reversed by LYC treatment (*p* < 0.05). These results suggest that LYC has the ability of promoting osteogenesis and suppressing adipogenesis through regulation of the FoxO1/PPARγ signaling in OVX rats.

## 4. Discussion

The disturbed redox homeostasis promotes adipogenesis and inhibits osteogenesis, thus attenuating bone formation [44,45]. The countermeasures focusing on alleviating oxidative stress may offer a novel solution for preventing the development of osteoporosis [46,47,48]. In the present study, the following evidence was provided using OVX rats and BMSCs: (1) LYC improves bone microarchitecture, mechanical strength and material constituents; (2) LYC increases serum P1NP levels, calcium nodules and bone GAGs levels, and decreases serum CTX-1 levels; (3) LYC decreases serum and/or bone marrow TC, TAGs, and LDL, increases serum HDL levels, and inhibits lipid droplets formation; (4) LYC attenuates ROS production and increases serum and bone marrow of T-AOC and SOD levels, and decreases serum MDA levels; (5) LYC increases the expression levels of FoxO1, OCN and Runx2, and decreases the expression levels of PPARγ and C/EBPα.

In the current study, LYC is demonstrated to reduce body weight gain and improve serum lipid metabolism, which is in line with the previous investigations [49,50]. In addition, we found that LYC is able to reduce lipid formation and improve lipid profiles in bone marrow. Moreover, LYC was reported to reduce lipogenesis in BMSCs [51]. It is known that osteoporosis may co-exist with dyslipidemia in postmenopausal women [52,53]. LYC was found to improve blood lipoprotein in postmenopausal women [53]. Thusly, these findings suggest that LYC may ameliorate lipid metabolism to improve bone quality in OVX rats.

In the present study, we found that LYC treatment did ameliorate bone quality evidenced by an improvement of bone microstructure, mechanical strength, and material profiles in OVX rats. This is in agreement with the previous investigations [54,55,56]. Our group also found that LYC improves bone quality in obese mice [23]. Clinically, LYC was reported to prevent osteoporotic bone loss in postmenopausal women [9,57]. Therefore, these results suggest that LYC has an ability to improve bone quality in osteoporotic patient, which may provide a novel strategy for the management of this prevalent degenerative bone disease.

In the current study, we first showed that LYC promotes osteogenesis in OVX rats by increasing the GAG contents and promoting calcium nodule formation in the femurs. Then, we found that LYC could attenuate the limitation of osteogenesis in BMSCs upon H_2_O_2_ stimulation. Likewise, LYC was reported to promote osteogenesis in OVX rats [55,56], BMSCs [58], and osteoporotic women [57]. Collectively, these results indicate that LYC may prevent the development of osteoporosis by promoting osteogenesis.

LYC is found to restore redox homeostasis by increasing serum and bone marrow levels of anti-oxidant markers (SOD and T-AOC), and decreasing serum levels of oxidant markers (MDA). In addition, LYC is demonstrated to reduce ROS production (DCFH-DA assay) in BMSCs upon H_2_O_2_ stimulation. Along this line, Iimura et al. [59] reported that LYC intervention could inhibit bone loss by reducing oxidative stress in OVX rats. The evidence from clinical studies also showed that LYC is able to decrease oxidative stress and inhibit bone resorption in postmenopausal women [27,60]. Together, our current findings in conjunction with the abovementioned findings from other groups suggest that LYC may prevent the development of osteoporosis through inhibition of oxidative stress overproduction.

The present study demonstrated an increase in the expressions of FoxO1, Runx2 and OCN, and a decrease in the expressions of PPARγ and C/EBPα in the femurs and tibias of OVX rats in response to the LYC treatment. These alterations were also demonstrated in BMSCs upon H_2_O_2_ stimulation. Similarly, LYC was reported to attenuate oxidative stress through upregulation of FoxO1 expression in mouse exposure to atrazine [61,62]. Additionally, LYC was reported to inhibit ROS overproduction and PPARγ expression in the hearts, kidneys, and livers of the rats on high-fat or high-cholesterol diets [63,64]. Liao et.al also reported that FoxO1 deficiency may promote bone loss through increasing ROS overproduction [65]. FoxO1 could facilitate osteoblasts differentiation and mannerization by inhibiting oxidative stress [66]. Moreover, FoxO1 may bind with the PPARγ promoter to inhibit the transcriptional activity of PPARγ, thus limiting the adipose differentiation [67,68]. Furthermore, a recent study reported by Ardawi et al. suggested that LYC promoted osteogenesis and inhibited adipogenesis in rat BMSCs [58]. Here, we also demonstrated that LYC increases calcium nodule formation and decreases lipid droplets formation in BMSCs and OVX rats. Using a FoxO1 inhibitor, we found that the actions of LYC on osteogenesis and adipogenesis were associated with the FoxO1 and PPARγ. Taken together, these findings indicate that LYC might promote osteogenesis and reduce adipogenesis by regulating redox homeostasis via the FoxO1/PPARγ signaling pathway in OVX rats.

In the current study, while the focus is on the beneficial effects of LYC in preventing osteoporosis through the regulation of redox homeostasis, it is essential to acknowledge the potential influence of confounding factors on the results. Lycopene is a strong antioxidant compound with an open-polyene chain [69]. This unique structure makes the compound possess strong hydrophobicity and chemical instability, thereby displaying a low bioavailability after ingestion [70]. Therefore, a ready-to-use approach was used to avoid the oxidation of LYC during storage in the present study. In addition, sunflower oil and DMSO were used as solvents during the experiments.

Some limitations still existed in the present study when interpreting the data. Firstly, we did not employ FoxO1-deficient mouse to investigate the action of LYC on bone quality. However, FoxO1 was reported to be positively involved in promotion of osteogenesis and inhibition of adipogenesis in OVX animals [71,72] and BMSCs [73]. LYC was able to attenuate oxidative stress via increasing FoxO1 expression in mice exposed to atrazine [61]. Secondly, we did not study the direct effect of LYC on PPARγ expression in BMSCs. However, we employ a FoxO1 inhibitor to show that LYC could inhibit PPARγ expression in BMSCs upon H_2_O_2_ stimulation. In addition, LYC was reported to inhibit PPARγ expression in hypercholesterolemic and obese rats [63,64].

## 5. Conclusions

In summary, LYC may attenuate bone loss through the promotion of osteogenesis and inhibition of adipogenesis via the regulation of redox homeostasis in OVX rats. The underlying mechanism behind these alterations may be related to the action of this compound on the FoxO1/PPARγ signaling. These results suggest that dietary consumption of LYC may offer a novel therapeutic strategy for the treatment of osteoporosis, which needs to be further identified in clinical trials. However, in future investigations, a FoxO1-deficient mouse should be employed to further elucidate the mechanism of the actions of LYC on bone quality.

## Data Availability

The raw data supporting the conclusions of this article will be made available by the authors on request.

## Abbreviations

BMSCs	Bone marrow mesenchymal stem cells
BMD	Bone mineral density
BS/TV	Bone surface/total volume
BV/TV	Bone volume/total volume
C/EBPα	CCAAT/enhancer binding proteins α
Conn.D	Connectivity density
CTX-1	C-terminal cross-linked telopeptide of type I collagen
EV	Estradiol valerate
FoxO1	Forkhead Box Protein O1
GAGs	Glycosaminoglycans
HDL	High-density lipoprotein
H&E	Hematoxylin & Eosin
LDL	Low-density lipoprotein
LYC	Lycopene
MDA	Malondialdehyde
OCN	Osteocalcin
ORO	Oil Red O
OVX	Ovariectomized
PINP	N-terminal propeptide of type1 procollagen
PPARγ	Peroxisome Proliferator Activated Receptor γ
ROS	Reactive oxygen species
Runx2	Runt-related transcription factor 2
SMI	Structure Model Index
SOD	Superoxide dismutase
TAGs	Triacylglycerols
T-AOC	Total antioxidant capacity
Tb.N	Trabecular number
Tb.Sp	Trabecular separation
Tb.Th	Trabecular thickness
TC	Total cholesterol
